# Single-Molecule Conductance of Non-Redox Proteins: Mechanisms, Measurements, and Applications

**DOI:** 10.3390/biom16040495

**Published:** 2026-03-25

**Authors:** Zhimin Fan, Miao Chen, Jie Xiang, Bintian Zhang

**Affiliations:** Shenzhen Key Laboratory of Precision Measurement and Early Warning Technology for Urban Environmental Health Risks, School of Environmental Science and Engineering, Southern University of Science and Technology (SUSTech), Shenzhen 518055, China

**Keywords:** protein conductance, non-redox proteins, charge transport mechanisms, single-molecule detection, bioelectronics

## Abstract

Charge transport underpins essential biological processes, including cellular respiration, photosynthesis, and enzymatic catalysis. Advances in molecular electronics have enabled single-molecule measurements that unequivocally establish redox-active proteins as efficient electron conductors, with their metal cofactors serving as intrinsic redox relays. By contrast, ubiquitous non-redox proteins lacking such redox centers have long been considered poor conductors. However, recent research has challenged this view, demonstrating that efficient charge transport in non-redox proteins can be mediated through polypeptide backbones, aromatic side-chain arrays, and hydrogen bond networks. This review surveys progress in understanding the single-molecule conductance of non-redox proteins. Firstly, we elucidate the fundamental transport mechanisms, highlighting the interplay between coherent tunneling and thermally activated hopping. We then provide an overview of state-of-the-art experimental techniques for single-molecule characterization. Through analysis of diverse systems spanning short peptides to large enzymes, we illustrate how aromatic amino acid networks and dynamic conformational fluctuations govern conductance, enabling emerging applications in label-free biosensing and single-molecule protein/DNA sequencing. Finally, we discuss persistent challenges and outline future opportunities for integrating protein-based conductors into bioelectronic devices. This review aims to stimulate further research and pave the way for novel applications harnessing protein conductance.

## 1. Introduction

Charge transport is fundamental to vital biological processes, such as photosynthesis and cellular respiration, and represents a core function that molecular electronics aims to emulate [1,2,3]. As conventional silicon-based electronics approach physical scaling limits, devices based on biomolecules have garnered significant attention due to their biocompatibility and functional diversity [4]. Among various biomaterials, proteins offer distinct advantages over traditional organic molecules, attributable to their atomically precise three-dimensional structures, genetically encoded self-assembly capabilities, and dynamic conformational gating in response to environmental stimuli (e.g., pH, light, and ligands) [5,6]. Elucidating charge transport in these complex soft materials is therefore crucial for advancing high-sensitivity biosensors and bioelectronic logic devices.

Early studies of protein conductance focused predominantly on redox-active proteins, such as Azurin and Cytochrome c [7]. Possessing intrinsic metal centers or cofactors, these proteins utilize these moieties as localized charge reservoirs and intermediate stepping stones, facilitating long-range electron transport via an incoherent hopping mechanism. While this cofactor-mediated transport is well-established by single-molecule experiments and consistent with Marcus theory, this cofactor-centric paradigm often overlooks the intrinsic electrical properties of the polypeptide backbone [8]. Furthermore, as redox activity is restricted to a small fraction of the proteome, relying solely on these proteins limits the scalability and universality of protein-based bioelectronics.

In contrast, the vast majority of naturally occurring proteins, including antibodies, structural proteins, and enzymes, lack metal cofactors and are collectively termed non-redox proteins. Traditionally, these proteins were considered poor electrical conductors due to the absence of low-ionization-energy metal centers and the substantial HOMO–LUMO energy gap of the amino acid backbone [7,9]. However, current evidence demonstrates that, even without cofactor assistance, these proteins support significant charge transport mediated by the peptide backbone, hydrogen bond networks, and aromatic side-chain arrays. Mechanistically, this transport is governed by a complex interplay between off-resonant tunneling, hopping via aromatic networks, and thermally activated flickering resonance. Notably, conductance in non-redox proteins is highly sensitive to thermal fluctuations and conformational dynamics, establishing a distinctive structure–conductance relationship [10]. This property opens up new possibilities for using non-redox proteins in bioelectronic devices, while also giving us a fresh perspective on how charges move through biological systems.

This review examines single-molecule conductance in non-redox proteins. We first outline the principal charge transport mechanisms and experimental device platforms. We then survey conductance properties across diverse non-redox systems and discuss emerging applications in label-free biosensing and single-molecule protein sequencing. Finally, we discuss key challenges in theoretical modeling and at electrode–protein interfaces, and we highlight opportunities to integrate proteins into functional electronic devices.

## 2. Mechanisms of Protein Conductance

Proteins, characterized by intricate folded structures and dynamic responsiveness to external stimuli (e.g., pH, ligands, and heat), possess immense potential as active components in next-generation bioelectronics. Unlike rigid solid-state materials, proteins are dynamic soft matter, with conformational fluctuations that profoundly influence charge propagation [11]. Understanding charge transport through proteins remains a central challenge in bioelectronics.

**Figure 1:**
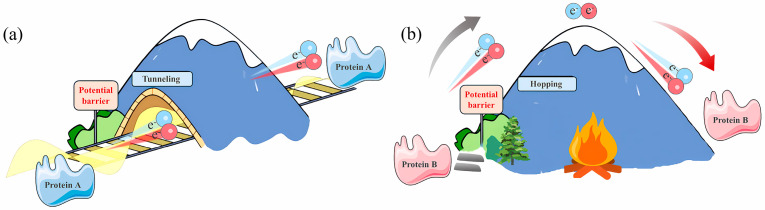
General mechanisms of protein conductance. (**a**) Protein electron tunneling: Electrons utilize their quantum wave nature to traverse energy barriers within the protein via a single-step transfer process, without thermal activation. (**b**) Protein electron hopping: Electrons leverage thermal conformational fluctuations to undergo sequential, thermally activated hops between localized redox sites or residues, enabling multi-step migration. (Adapted from Ref. [12], ACS).

This section provides a comprehensive analysis of charge transport in single-molecule protein junctions. We broadly categorize conductance into two dominant regimes, coherent tunneling and incoherent hopping (Figure 1 and Table 1), noting that the boundary between them is often blurred by thermal fluctuations, giving rise to hybrid behaviors. We first summarize coherent tunneling, including the role of secondary structures in modulating the decay constant (*β*) and the dual-pathway model in redox proteins. We then discuss thermally activated hopping, extending the analysis to flickering resonance and anomalous length-dependent behaviors observed in non-redox systems, where structural dynamics and electronic coupling interplay critically.

### 2.1. Coherent Tunneling

In nanoscale biomolecular junctions with short effective transport lengths (typically *L* < 1–2 nm, depending on the protein structure and temperature), charge transport is often dominated by coherent tunneling [13]. Fundamentally, in the ideal coherent tunneling limit, the electron does not thermally relax onto any specific amino acid or cofactor site; instead, the electron wavefunction penetrates the potential barrier formed by the protein via virtual intermediate states (Figure 1a). It is important to note, however, that virtual occupation does not imply zero probability amplitude on intermediate sites. In systems with strong electronic coupling or periodic structures (e.g., DNA base stacks or aromatic-rich peptides), intermediate moieties can exhibit finite, albeit short-lived, charge occupation probability [14]. This phenomenon, sometimes described as partial delocalization or band-like transport, blurs the strict distinction between tunneling and hopping. Nevertheless, in the canonical tunneling regime where conductance decays exponentially with distance:

G(L)=Aexp(−βL)

where *G*(*L*) is the single-molecule conductance measured at an electrode separation distance *L*, *A* is the contact conductance that depends on the metal-molecule coupling at the electrodes, and *β* is the decay constant (units of Å^−1^) that characterizes the efficiency of electron tunneling through the protein medium [15]. A distinguishing feature of this mechanism is its weak temperature dependence and transport occurs without full thermal relaxation of the charge carrier onto intermediate sites, distinguishing it from the sequential, thermally activated hops of incoherent transport. Unlike the strong Arrhenius activation observed in hopping, tunneling is an inherently ultrafast quantum process. However, in soft biological materials, it is not strictly temperature independent [16]. Thermal vibrations modulate the distance and orientation between amino acid residues (dynamic disorder), causing fluctuations in the electronic coupling term (H_DA_) [16]. The electronic coupling H_DA_ describes the strength of quantum mechanical interaction between donor and acceptor states; it depends exponentially on the through-space or through-bond separation and is modulated by the orientation of the interacting orbitals and the intervening protein matrix. Stronger coupling facilitates more efficient electron tunneling. Consequently, tunneling conductance may exhibit a mild temperature dependence, distinct from the exponential activation characteristic of hopping.

#### 2.1.1. Tunneling in Redox Proteins

In redox proteins containing metal centers (e.g., Cu, Fe), intuition might suggest that electrons utilize these centers as stepping stones. However, extensive experiments at low temperatures and short distances have demonstrated that tunneling remains the dominant mechanism [17]. Importantly, the metal center often acts not as a serial relay, but rather as a sophisticated gate.

The Azurin represents a prime example. Conductance measurements down to 4 K reveal a temperature-independent current, confirming transport via off-resonant tunneling through the protein backbone’s energy levels [18]. To explain the significant conductance modulation by the copper center, a dual-channel model has been proposed. In this framework, Channel I represents faster background tunneling through the peptide backbone and hydrogen-bond network, while Channel II involves a slower pathway influenced by the copper center. The metal center does not necessarily conduct the bulk of the current directly. Instead, its oxidation state (Cu^2+^/Cu^+^) generates an electrostatic field that perturbs the energy alignment of the surrounding backbone orbitals. This effect mimics a transistor gate: by modifying the effective tunneling barrier height through electrostatic (capacitive) coupling, the metal center regulates the total conductance [19].

Additionally, research on Cytochrome c underscores the pivotal role of molecular orientation. Efficient coupling into the superexchange pathway occurs only when the heme edge is covalently pinned or anchored proximal to the electrode. In contrast, random orientation arising from non-specific adsorption interposes an insulating protein shell between the cofactor and the electrode, creating an additional dielectric barrier that significantly suppresses tunneling efficiency.

#### 2.1.2. Tunneling in Non-Redox Proteins

At short distances, non-redox proteins exhibit remarkable tunneling efficiencies, with decay constants (*β* ≈ 0.1–0.6 Å^−1^) significantly lower than those of saturated alkane chains (~1.0 Å^−1^) [2]. This high efficiency originates from π-orbital delocalization along the peptide backbone, a process strongly modulated by secondary structure. Comparative studies indicate that β-sheets generally exhibit lower attenuation factors than α-helices. This difference arises because β-sheets possess greater structural rigidity and feature axially ordered hydrogen-bond networks, creating anisotropic channels conducive to electron wavefunction delocalization [20]. This structural feature explains why amyloid fibrils, composed of stacked β-sheets, can function as robust bio-nanowires supporting long-range coherent transport.

Beyond the backbone, side-chain chemistry plays a critical auxiliary role. Electron-rich aromatic side chains (e.g., tryptophan, Trp) can significantly lower the tunneling barrier. For instance, poly-Trp sequences exhibit much higher conductance than poly-Ala, as spatial overlap of aromatic π-orbitals mediates efficient superexchange [21]. More remarkably, in non-redox proteins such as streptavidin, resonant tunneling peaks have been observed at specific bias voltages [8]. This phenomenon is attributed to energy-level renormalization induced by the unique electrostatic environment of the folded protein, which can shift internal frontier orbitals into alignment with the electrode Fermi level. These findings suggest that through precise control of folding and electrostatic fields, even non-redox proteins can achieve highly efficient electron tunneling.

Notably, in systems with strong electronic coupling, such as periodic DNA base stacks or densely packed aromatic peptide arrays, intermediate sites can exhibit finite charge occupation probability, leading to behaviors that bridge the tunneling and hopping regimes. This phenomenon, sometimes described as ‘band-like’ transport or ‘partially delocalized’ tunneling, has been observed in guanine-rich DNA sequences and synthetic aromatic foldamers [14]. In such cases, the strict dichotomy between tunneling and hopping becomes insufficient, and more sophisticated models incorporating quantum coherence and dynamic disorder are required.

### 2.2. Incoherent Hopping

Beyond the distance regime where coherent tunneling dominates (typically >1– 2 nm for most proteins at room temperature) or at elevated temperatures that promote thermal activation, the electron wavefunction loses phase coherence due to environmental coupling. In this regime, the dominant mechanism shifts to thermally activated incoherent hopping (Figure 1b) [22]. Experimentally, temperature-dependent measurements serve as the primary method for distinguishing these regimes: coherent tunneling exhibits weak temperature dependence (often with small negative or positive coefficients due to thermal expansion/contraction effects), while hopping shows strong Arrhenius-type activation [23].

It is important to note that the crossover distance is not universal—it depends on the protein’s secondary structure, the density of aromatic residues that can serve as transient hopping sites, and the operating temperature. For example, proteins with dense aromatic networks may extend the coherent tunneling regime, while flexible structures with sparse aromatic content may transition to hopping at shorter distances [13]. Electrons transiently occupy localized sites (cofactors or amino acid residues), undergoing sequential redox events and vibrational relaxation. Macroscopically, conductance scales inversely with length (G∝1/L), enabling efficient long-range transfer in contrast to tunneling’s exponential decay [15]. The hopping rate *K*_ET_ exhibits an Arrhenius-like relationship, following Marcus semiclassical theory for non-adiabatic electron transfer [24]:

$$K_{\mathrm{ET}}=\frac{2\pi }{\hbar }|V{|}^{2}\frac{1}{\sqrt{4\pi \lambda k_{B}T}}exp\left[-\frac{{\left(\Delta G^{0}+\lambda \right)}^{2}}{4\lambda k_{B}T}\right]$$

where *V* is the electronic coupling between donor and acceptor states, *λ* the reorganization energy, and Δ*G*^0^ the driving force. The electronic coupling *V* depends exponentially on the donor–acceptor separation and is modulated by the orientation of redox centers and the intervening protein medium. Protein systems typically operate in the non-adiabatic regime because redox centers or aromatic residues are separated by distances (typically 5–10 Å). Moreover, the protein interior is characterized by a low dielectric constant, resulting in extremely weak direct orbital overlap between donors and acceptors. Consequently, these systems exhibit weak electronic coupling (*V* < *k_B_T*). In this regime, the electron transfer event is rate-limited by the probability of tunneling through the barrier, and the rate scales with |*V*|^2^ as described by Fermi’s rule.

#### 2.2.1. Hopping in Redox Proteins

In natural systems like respiratory chains and photosynthetic reaction centers, evolution has optimized the use of metal cofactors as stepping stones to overcome energy barriers for long-distance transport. Iron-sulfur (Fe-S) proteins are paradigmatic examples [25]. In systems such as ferredoxin, multiple Fe-S clusters are precisely arranged with spacings of 10–15 Å, forming a quasi-periodic array of redox sites. Within this architecture, an electron executes sequential multi-step hopping between metal clusters. Each individual hop is effectively a short-range tunneling event; this segmented transport strategy drastically lowers the effective barrier per step, enabling electrons to traverse tens of nanometers. Single-molecule studies of Fe-S protein complexes reveal the Marcus inverted region, where current saturates or declines at high bias, confirming hopping as the rate-limiting process.

#### 2.2.2. Hopping in Non-Redox Proteins

In the absence of metal centers, non-redox proteins utilize specific amino acid residues (primarily oxidizable tyrosine or tryptophan) as transient charge carriers [26]. While the high aqueous reorganization energy of amino acids (*λ* ≈ 1–2 eV) typically impedes transport, the protein’s hydrophobic core can shield charge from solvent, reducing λ to ~0.2–0.5 eV and enabling hopping [26,27,28]. Observed behaviors, however, often deviate from classical models, prompting new theoretical frameworks.

The flickering resonance (FR) model, proposed by Beratan and Skourtis, can be viewed as a hybrid of tunnelling and hopping [16,29]. This mechanism suggests that thermal fluctuations can transiently align the energy levels of multiple aromatic residues, thereby allowing for electron transport via a quasi-coherent, ballistic-like mechanism within brief resonance windows. This perspective highlights the potential active role of conformational dynamics in facilitating, rather than always hindering, long-range electron transfer. The applicability of FR, however, is subject to strict energetic and structural criteria: the electronic coupling (*V*) between sites must be comparable to the thermal energy (*k_B_T*). In many globular non-redox proteins, aromatic residues are sparse, leading to weak coupling (*V* ≪ *k_B_T*) that favors standard hopping over resonance [13]. Therefore, FR is likely restricted to specific systems with dense, well-coupled π-stacking networks, such as certain DNA-protein assemblies or specialized bacterial pili (e.g., *Geobacter* PilA), rather than representing a universal mechanism [30].

**Table 1:** Comparison of Different Protein Conductance Mechanisms.

Mechanism	Key Features	Typical Proteins	Ref.
Coherent Tunneling	Weak temperature dependence Exponential distance decay Phase-coherent quantum transport Typically off-resonant	Azurin Cytochrome b562 α-helical peptides Modified myoglobin	[9,11,31,32,33,34]
Hopping	Strong temperature dependence Shallow distance dependence Via real intermediate states Thermally activated	Linear multi-heme chains Cytochrome nanowires Outer-membrane cytochromes Soluble tetraheme model protein	[35,36,37,38]
Flickering resonance	Temperature-dependent Exponential distance decay Transient energy alignment via dynamics	OmcS filaments in Geobacter Flexible, disordered heme chains Conformationally dynamic multi-heme proteins	[16,39,40,41]
Superexchange	Weak temperature dependence Strong exponential distance decay Virtual-state-mediated tunneling	Bacterial photosynthetic reaction centers Iron-sulfur proteins	[42,43]

Additional anomalies include quadratic length dependence ($R\propto L^{2}$) observed in consensus tetratricopeptide repeat (CTPR) proteins, suggestive of diffusive rather than drift transport. Under high bias, drift (R∝L) is expected; the observed scaling may reflect strong dielectric screening that nullifies internal fields or non-equilibrium injection limitations at interfaces [6]. These phenomena underscore the complex, non-equilibrium nature of transport in non-redox proteins and the need for advanced theoretical models integrating structural dynamics and statistical physics.

## 3. Single-Molecule Protein Conductance Measurement Techniques

As quintessential soft matter, the electrical properties of proteins are highly susceptible to perturbations arising from anchoring strategies, electrode contact geometries, solvent environments, and external electric fields. Consequently, characterization techniques must not only exhibit high sensitivity but also rigorously account for and mitigate experimental artifacts [44]. While traditional macroscopic ensemble measurements often obscure informative stochastic fluctuations, single-molecule techniques provide a direct window into charge transport dynamics. However, distinguishing intrinsic electronic signals from confounding factors such as ionic leakage currents, mechanical deformation, and contact instabilities remains the central experimental challenge in the field [45].

**Figure 2:**
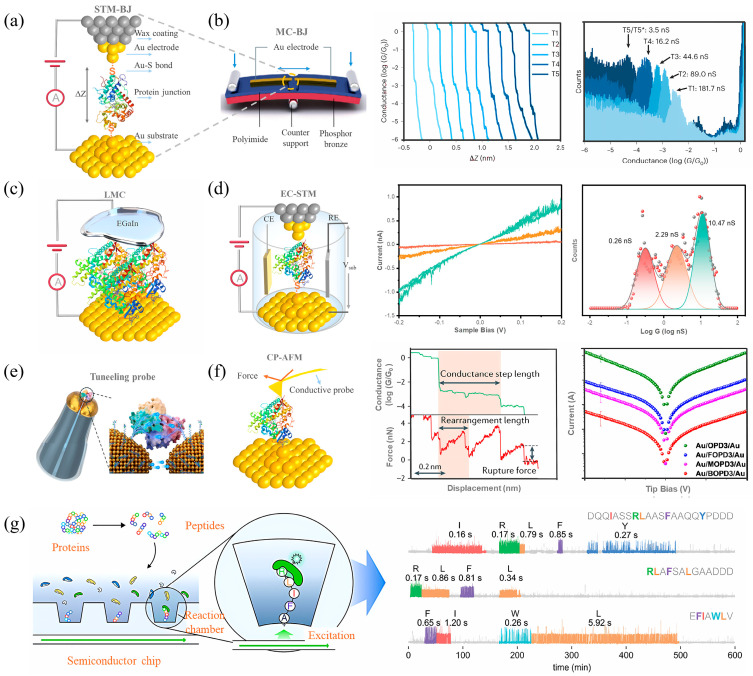
Platforms for single-molecule protein conductance measurements. (**a**) Schematic of a scanning tunneling microscope-based break junction (STM-BJ) measurement. (**b**) Schematic of a mechanically controlled break junction (MCBJ) measurement (left), along with 2D (middle) and 1D (right) histograms related to protein conductance measured by STM-BJ or MC-BJ. (**c**) Schematic of soft-contact conductance measurements using liquid metal eutectic gallium-indium (EGaIn). (**d**) Schematic of an electrochemical scanning tunneling microscope (EC-STM) (left), accompanied by protein conductance-associated I-V curves (middle) and histograms (right). (**e**) Schematic of a single streptavidin protein-coupled quantum mechanical tunneling (QMT) probe. (**f**) Schematic illustration of a conductive atomic force microscope (CP-AFM) (left) for simultaneous single-molecule measurements (middle) of current (green) and force (red), semilogarithmic averaged I-V curves of single-molecule (right). (**g**) Real-time detection of N-terminal amino acids (NAA) in nanoscale reaction chambers on a semiconductor chip (left), employing dye-labeled NAA recognizers, and dynamic sequencing of the synthetic peptides (right). Panel (**a**) is adapted from ref. [54], Springer Nature Limited. Panel (**b**,**f**) is adapted from ref. [59], Springer Nature Limited. Panel (**c**) is adapted from ref. [28], ACS. Panel (**d**) is adapted from ref. [53], ACS. Panel (**e**) is adapted from ref. [60], AAAS. Panel (**g**) is adapted from ref. [61], AAAS.

This section elucidates the core experimental techniques employed to probe protein conductance at the single-molecule level. To provide a clear methodological framework, we structure the discussion around three primary paradigms that address distinct scientific questions (Figure 2 and Table 2): (i) scanning probe-based break junction techniques for the statistical acquisition of single-molecule conductance; (ii) physical contact-based techniques for investigating mechano-electric coupling and dynamic processes; and (iii) integration and validation-oriented techniques that bridge fundamental characterization and applied device metrics. Drawing upon the latest data, we critically analyze the working principles, representative applications, and inherent limitations of each method.

### 3.1. Scanning Probe-Based Break Junction Techniques: STM-BJ, EC-STM and MCBJ

These techniques statistically determine single-molecule conductance by repeatedly forming and breaking molecular junctions between electrodes. Their core strength lies in generating rich statistical data on junction formation probability, conductance value distributions, and configurational diversity.

#### 3.1.1. Scanning Tunneling Microscope Break Junction (STM-BJ)

STM-BJ leverages the Ångström-level spatial resolution and picoampere current sensitivity of STM. A key advantage is its operation in near-physiological aqueous environments, which is critical for preserving native protein conformations [46]. However, STM-BJ data typically exhibit significant dispersion due to junction heterogeneity: variations in protein adsorption conformations, microscopic differences in metal-molecule linkage geometry, and intrinsic protein conformational fluctuations.

In a typical measurement, the STM tip performs repeated approach–retract cycles over a substrate functionalized with target proteins (Figure 2a). Upon retraction, a single molecule may be trapped between the electrodes. A stable molecular junction is signified by a characteristic conductance plateau in the recorded trace, the value of which represents the single-molecule conductance [47]. Statistical analysis of thousands of traces, compiled into 1D or 2D conductance histograms, yields the most probable conductance value. While conventional 1D histogram analysis can introduce user-defined selection bias, recent advances employ unsupervised machine learning to objectively classify conductance behaviors and distinguish true molecular junctions from artifacts [48].

#### 3.1.2. Electrochemical Scanning Tunneling Microscopy (EC-STM)

EC-STM represents a powerful functional extension of the STM platform, integrating a bipotentiostat to enable independent control of the tip and substrate potentials versus a reference electrode (Figure 2d). This configuration is indispensable for investigating redox-active proteins and the influence of electrostatic potentials on charge transport [49]. The core capability is electrochemical gating: by precisely tuning the electrode’s Fermi level, researchers can align it with the protein’s frontier molecular orbitals (HOMO/LUMO), thereby modulating the charge injection barrier and transport efficiency in a manner analogous to a field-effect transistor. This allows for the direct probing of redox states and potential-dependent conductance switching [50]. A major technical challenge in liquid environments is distinguishing faradaic ionic leakage currents from the electronic tunneling current of interest. Rigorous controls, including measurements in protein-free buffers and using well-insulated tips, are prerequisite for validating electronic conductance, directly addressing the experimental question of whether transport is “indeed electronic or do ions contribute.”

#### 3.1.3. Mechanically Controllable Break Junction (MCBJ)

Although sharing the break-junction paradigm, MCBJ differs markedly in its preference for non-aqueous environments. The MCBJ technique employs a microfabricated, freestanding metallic bridge on a flexible substrate (Figure 2b). A piezoelectric actuator bends the substrate, stretching and ultimately fracturing the bridge to create a pair of electrodes with sub-nanometer separation control [51]. This design offers exceptional mechanical stability and negligible drift, making it ideally suited for operation in cryogenic or vacuum environments and for probing conformational dynamics over extended periods. Unlike the transient nature of STM-BJ, MCBJ allows researchers to park the electrodes at a fixed separation, facilitating real-time observation of molecules switching between metastable states—a valuable window into folding/unfolding kinetics. A primary limitation is that typical vacuum/cryogenic operation may dehydrate proteins, altering native structures compared to liquid-phase techniques.

#### 3.1.4. Applications and Scientific Insights

Scanning probe-based break junction techniques (STM-BJ, EC-STM, and MCBJ) collectively provide versatile platforms for statistical determination of single-molecule protein conductance, spanning aqueous physiological conditions (STM-BJ/EC-STM), electrochemical gating (EC-STM), and high-stability cryogenic/vacuum environments (MCBJ). These methods demonstrate the scale-dependent nature of orientation effects, conformational dynamics, and structure-conductance relationships, ranging from amino acids to large macromolecules, and underscore the critical role of contact geometry and environmental factors.

Mechanistic Insights and Orientation-Dependent Transport: Comparative STM-BJ studies on azurin have demonstrated that site-specific anchoring (e.g., via the N42C cysteine mutant) enables reversible, bias-induced conductance switching, a feature absent in randomly oriented wild-type proteins [19]. This conclusively establishes that molecular orientation governs both tunneling barrier width and electronic coupling strength. In non-redox systems, STM measurements of thermophilic archaeal proteasomes (conductance ~ 2.69 nS) have underscored the pivotal role of aromatic residues in facilitating efficient tunneling, revealing how π-stacking networks lower decay constants. Extending to electrochemical control, EC-STM has shown that long-range conductance is modulated by ligand-mediated contact chemistry in the electrolyte, imparting pathway selectivity [52]. Furthermore, EC-STM mapping of streptavidin has revealed how contact geometry and surface charge orchestrate conductance distributions in bioelectronic circuits [53].

Conformational Dynamics and State-Specific Signatures: STM-BJ has enabled real-time monitoring of functional dynamics, such as distinguishing discrete catalytic states in formate dehydrogenase through unique conductance signatures [54]. In MCBJ, exceptional junction stability facilitates observation of telegraph noise, providing kinetic insights into folding/unfolding processes.

Molecular Fingerprinting and Length/Rigidity Effects: MCBJ has proven particularly powerful for building high-precision conductance atlases. Studies have distinguished tunneling currents among 12 amino acids and phosphorylated tyrosine, laying a quantitative foundation for partial peptide sequencing and post-translational modification analysis [55]. Complementary libraries for 10 amino acids and dimethionine have enabled unambiguous identification [56]. At the peptide scale, alanine series measurements revealed counterintuitive behavior: at short electrode separations, flexible longer chains (tetra-alanine) exhibit higher conductance than rigid shorter ones (di-alanine) due to folding into metastable configurations that enhance superexchange or hopping pathways. Conductance reverts to exponential decay upon full stretching, highlighting non-trivial rigidity-length dependencies resolved via machine learning classification [57].

Macromolecular and Nanowire Characterization: These techniques can be extended to complex systems. MCBJ measurements of bacterial pilin (PilV) and lysozyme have captured characteristic conductance steps (~0.2 *G*_0_ for PilV), distinguishing protein-bridged junctions from metallic quantum point contacts [58].

These exemplary applications demonstrate the complementary strengths of break-junction methods in resolving mechanistic debates and enabling emerging sensing paradigms, particularly in non-redox proteins where aromatic networks and conformational gating dominate transport.

### 3.2. Physical Contact-Based Measurement Techniques: CP-AFM and Tunneling Probes

These techniques rely on establishing stable physical or tunneling contact between an electrode and the sample, enabling spatially fixed, long-duration measurements ideal for studying force modulation and dynamic processes. 

#### 3.2.1. Conducting Probe Atomic Force Microscopy (CP-AFM)

CP-AFM addresses the mechanical control gap inherent to STM. Using a conductive cantilever tip, it operates in contact mode, enabling the simultaneous acquisition of topography and current while precisely regulating the applied mechanical force (Figure 2f). This allows for direct investigation of mechano-electric coupling effects, where mechanical compression can shorten tunneling distances or reorganize internal hydrogen-bond networks, potentially inducing a transition in transport mechanism [62]. CP-AFM is also uniquely suited for characterizing extended structures like self-assembled protein nanowires (e.g., amyloid fibrils), providing uninterrupted conductivity mapping along their axis—a capability inaccessible to break-junction techniques [63].

#### 3.2.2. Functional Recognition Tunneling Probe Measurements

These techniques utilize fixed nanogap electrodes or functionalized probes (Figure 2e). Distinct from break-junction methods that rely on repetitive rupture cycles, tunneling probe measurement utilizes a spatially fixed gap or a feedback-controlled probe to facilitate molecular identification and fingerprinting [64]. By prioritizing the analysis of time-resolved current fluctuations over statistical counting, this approach enables the continuous observation of single-molecule dynamics over extended timescales (ranging from milliseconds to seconds). The core strategy involves the functionalization of electrodes with specific recognition agents (e.g., antibodies, ligands, or hydrogen bond donors/acceptors). Upon the diffusion of a target protein into the gap and its subsequent specific binding, a bridged molecular junction is formed (Electrode-Recognition Molecule-Protein-Recognition Molecule-Electrode). In this state, the tunneling current exhibits characteristic telegraph noise. The amplitude and frequency of these fluctuations encode the chemical fingerprint of the molecule, providing a dynamic basis for high-specificity single-molecule identification [65,66].

#### 3.2.3. Applications and Scientific Insights

In contrast to the statistical, junction-breaking approach of scanning probe methods, physical contact-based techniques like CP-AFM and functional tunneling probes offer a distinct paradigm centered on stable, spatially resolved interrogation. Their defining capability is the precise application and measurement of mechanical force (CP-AFM) or the long-duration monitoring of fixed molecular junctions (tunneling probes). This enables unique investigations into how pressure, deformation, and dynamic binding events directly modulate or report on charge transport, addressing questions inaccessible to transient break-junction measurements [67]. The following applications highlight how these methods elucidate mechano-electric coupling, validate transport pathways, and enable next-generation biosensing architectures.

Mechano-Electric Coupling and Transport Modulation: CP-AFM studies directly probe how mechanical stress perturbs electronic structure. Research on Azurin revealed that its metal center confers significant mechanosensitivity: under combined high temperature and pressure, the holo-protein undergoes a structural transition that shifts electron transport from tunneling to thermally activated hopping [33]. In contrast, the metal-free apo-form behaves like a simple compressible dielectric, highlighting the specific role of the redox cofactor in transducing mechanical force into an electronic response [68].

Contact Mechanics and Effective Transport Distance: A critical contribution of CP-AFM is in elucidating contact physics. A key methodological consideration is distinguishing between elastic compression and physical penetration of the insulating protein shell. Studies on designed CTPR proteins identified a critical force threshold (~50 nN) for observing significant conductance in longer constructs, suggesting that high loads may allow the probe to contact a conductive core, which is vital for defining the true effective transport distance [69]. This relates directly to the experimental discrepancy, where significant conductance in some proteins was only observed after applying high force in CP-AFM [6].

Characterization of Extended Bio-Nanostructures: CP-AFM’s dual imaging capability is indispensable for characterizing self-assembled protein nanostructures. For instance, continuous conductance mapping along elastin-like polypeptide fibrils confirmed their significant intrinsic solid-state conductivity [70]. This, combined with their exceptional thermal stability, establishes their potential as robust, self-assembled biological nanowires, a finding that bridges single-molecule measurements and macroscopic material properties.

Dynamic Conformational Readout via Functionalized Probes: Tunneling probe measurements excel at monitoring dynamics. Using the streptavidin-biotin system as a paradigm, functionalizing a probe tip with biotin allows the capture of a single protein, inducing characteristic multi-level current switching [1]. Analyzing the lifetimes and transition probabilities of these states from long-duration time traces elucidates the protein’s dynamic conformational equilibrium under an electric field, proving that quantum tunneling can sensitively read out microsecond-scale fluctuations [60,71].

Innovation in Integrated Sensing Devices: The development of quantum tunneling nanopipettes represents a significant fabrication advance. These are created via feedback-controlled electrochemical etching of double-barreled capillaries, resulting in a tip with an integrated nanogap and microfluidic channel [72]. This monolithic “fluidics-electronics” design is exceptionally suited for trapping and detecting single proteins in solution, paving the way for portable biosensors.

In summary, CP-AFM and functional tunneling probes provide a complementary and crucial perspective to break-junction methods. They uniquely quantify how mechanical forces gate or alter transport mechanisms, rigorously define effective transport pathways by clarifying contact mechanics, and enable continuous, high-temporal-resolution observation of molecular dynamics. These insights are fundamental for progressing from phenomenological conductance measurements toward a predictive understanding of charge transport in soft, dynamic biological matter and for the rational design of bio-electronic interfaces and devices.

### 3.3. Integration and Validation-Oriented Measurement Techniques

These techniques aim to bridge the gap between fundamental single-molecule characterization and macroscopic device applications or provide integrated solutions for specific applications like high-throughput analysis.

#### 3.3.1. Liquid Metal Contact (EGaIn) Conductance

To address potential physical penetration or structural damage from rigid metal electrodes (e.g., Au, Pt) on soft protein monolayers, EGaIn has emerged as a superior soft liquid-metal contact. This technique measures ensemble conductance across large-area protein self-assembled monolayers (SAMs) [73], bridging single-molecule studies and macroscopic bioelectronic devices (Figure 2e). A key feature is the rapid formation of an ultrathin (~0.7 nm) native gallium oxide (Ga_2_O_3_) skin, which provides rheological stability for conical droplet geometry while allowing efficient electron tunneling [74]. Typically, the EGaIn droplet forms a gentle conformal top contact on a protein SAM adsorbed to a bottom electrode, yielding Metal/Protein//Ga_2_O_3_/EGaIn junctions (//denotes physisorption). This architecture offers high yields, low shorting rates, and preservation of protein integrity. Although ensemble-based, current density analysis yields attenuation factors (β) consistent with single-molecule data. Consequently, EGaIn has been instrumental in validating long-range electron transport (>10 nm) in proteins, challenging conventional tunneling limits [75].

#### 3.3.2. Integrated Semiconductor Molecular Devices

Site-specific integration of single protein molecules into fixed nanogap electrodes is essential for high-performance semiconductor nanoelectronics and ultrasensitive biosensors (Figure 2g). Unlike traditional mechanically actuated techniques (e.g., STM or MCBJ), these platforms leverage scalable CMOS-compatible fabrication to create stable, fixed-gap architectures, enabling massively parallel monitoring of thousands of junctions on a single chip [76]. The primary advantage is exceptional scalability and temporal resolution (down to microseconds), facilitating real-time current tracing and machine learning-based analysis of amplitude, duration, and kinetics for precise fingerprinting of biological events.

The core molecular bridge strategy involves spanning the gap with a conductive wire (e.g., dsDNA or engineered peptide) to establish baseline conductance [77]. A target protein (e.g., polymerase or helicase) is then bioconjugated to the bridge. Its functional motions, such as substrate binding or conformational changes, perturb the bridge tension or electrostatics, thereby inducing field-effect gating that transduces these subtle dynamics into measurable conductance fluctuations.

#### 3.3.3. Applications and Scientific Insights

Moving beyond the fundamental characterization offered by single-point junction techniques, integration and validation-oriented methods provide critical insights at the device-relevant and systems level. EGaIn soft-contact measurements deliver statistically robust, ensemble-averaged data that bridge the gap to macroscopic electronics, while integrated semiconductor devices unlock scalable, high-throughput analytical capabilities [77]. The applications of these platforms converge on these key objectives: validating long-range transport phenomena, enabling the rational design of bioelectronic interfaces, and pioneering transformative analytical applications like single-molecule proteomics.

Validation of Long-Range Tunneling: A primary contribution of EGaIn technology is the rigorous validation of charge transport over biologically relevant distances. Studies on bacteriorhodopsin (bR) multilayers demonstrated an exceptionally low distance decay factor (*β*), suggesting that its ordered retinal chromophores and α-helical bundle create a highly efficient “electron-transparent” pathway [3]. Similarly, measurements on oriented Photosystem I (PSI) complexes confirmed temperature-independent, non-resonant tunneling over distances of ~9 nm [78]. These ensemble findings provide crucial, reproducible evidence that challenges the classical tunneling limit and supports the existence of efficient extended electronic states in ordered protein assemblies.

Platform for Rational Design and Interface Engineering: EGaIn serves as an indispensable testbed for the rational design of peptide-based electronic materials. Its gentle contact preserves monolayer integrity, allowing structure-function relationships to be cleanly probed. Research has demonstrated that strategic incorporation of redox-active heme cofactors can enhance current density by several orders of magnitude [28]. Furthermore, studies on site-specific doping, such as inserting histidine at defined positions within an oligo-alanine scaffold, have shown that single-atom changes can reshape the molecular energy landscape, thereby enabling precise, atomic-level control over conductance. This positions EGaIn as a key tool for iterative biomolecular design.

The Critical Role of Ensemble Validation: The ensemble nature of EGaIn provides a necessary counterpoint to single-molecule techniques by averaging over heterogeneous contact geometries. This capability is vital for distinguishing intrinsic molecular properties from junction-configuration artifacts [79]. A seminal example is the study of streptavidin-biotin binding: EGaIn measurements revealed that biotin binding does not significantly alter the average monolayer conductance, challenging interpretations derived solely from variable single-molecule STM data [79]. This highlights the necessity of combining single-molecule and ensemble approaches to build a complete and accurate picture of protein conductance.

The Potential of Integrated Devices for Single-Molecule Protein Sequencing: Integrated semiconductor molecular devices represent the frontier of applied protein electronics. Their core advantage is massive parallelism and microsecond temporal resolution. A groundbreaking application is the development of platforms for single-molecule peptide sequencing [61]. Engineers have created dynamic systems using N-terminal amino acid binders (NAABs), where the specific binding of an NAAB to a peptide terminus generates a characteristic current pulse [80]. As different amino acids yield unique electronic signatures, this approach, coupled with sequential chemistry, enables real-time peptide sequence reading. A key challenge remains the Debye screening limit in physiological buffers, necessitating operation in dilute solutions or innovative short-range sensing architectures to ensure efficient signal transduction [81].

In summary, EGaIn and integrated semiconductor devices address the critical translational gaps in protein electronics. EGaIn provides essential validation of long-range transport and serves as a high-fidelity platform for designer biomaterials, offering ensemble data that contextualize and ground single-molecule observations. Meanwhile, integrated devices demonstrate the scalable application of these principles, most notably in pioneering ultra-sensitive biosensing and sequencing technologies. Together, these approaches ensure that fundamental discoveries in single-molecule protein conductance can be rigorously validated and successfully transitioned towards practical bioelectronic technologies and tools.

These complementary paradigms, including statistical, mechano-sensitive, and device-oriented approaches, collectively resolve the experimental discrepancies in protein conductance and pave the way for robust bioelectronic applications.

**Table 2:** Comparison of Different Protein Conductance Measurement Methods.

Technique	Advantages	Disadvantages	Typical Measurement Conditions	Technical Features	Ref.
STM (EC-STM and STM-BJ)	High resolution Versatile environments Sensitive to electronic properties	Susceptible to interference Sample limitations Tip-induced artifacts	Molecular anchoring Room temperature Controlled potential vs. reference (EC-STM) Electrochemical environment (EC-STM) Vacuum/inert gas (STM-BJ)	Gap: Adjustable Molecules: Single Scalability: Low Reproducibility: Moderate	[8,82,83,84]
CP-AFM	Simultaneous topography and conductance mapping Applicable in solid/liquid phases	Sample or tip damage Lower electronic Complex data interpretation	Constant loading force Air, vacuum, or solution Room temperature	Gap: Contact Molecules: Ensemble Scalability: Low Reproducibility: High	[68,85,86,87,88]
MCBJ	Mechanical stability Low noise for statistical analysis Suitable for vacuum liquid environments	Limited environmental flexibility Potential gap drift over time	Mechanically controlled nanogap Repeated junction formation Low-noise current detection Vacuum or inert gas Room temperature or cryogenic	Gap: Adjustable Molecules: Single Scalability: Low Reproducibility: Moderate	[54,82,89,90,91]
Tunneling Probe	Stable long-term probing High sensitivity to conformational dynamics	Probe limitations Noise susceptibility	Nanogap Bias-dependent fluctuation analysis Liquid environment Room temperature	Gap: Fixed Molecules: Single Scalability: Low Reproducibility: Low	[60,72,92,93]
EGaIn Contact	Soft, deformable contacts Minimizing mechanical damage Reproducible Interface	Limited liquid holding capacity Potential toxicity in applications	Large-area junction Protein thin film Soft top electrode Ambient conditions Room temperature	Gap: Film thickness Molecules: Ensemble Scalability: High Reproducibility: High	[28,94,95,96]
Semiconductor Device	Scalable CMOS- compatible fabrication High-throughput potential via arrays	Noise sensitivity Costs for custom chips	Solid-state junction Lithographically defined electrodes Liquid or air environment Temperature-dependent transport	Gap: Fixed Molecules: Single/Ensemble Reproducibility: Moderate	[61,93,97]

## 4. Non-Redox Proteins and Conductance

While early research on protein conductance primarily focused on redox-active species, the vast majority of proteins in nature (including most enzymes, antibodies, receptors, and structural proteins) are classified as non-redox proteins [98]. Investigating these systems is pivotal for filling a critical gap in the biophysical landscape: elucidating how electrons achieve efficient cross-scale transport through polypeptide backbones and supramolecular networks in the absence of metal centers acting as energetic stepping stones [11]. Beyond fundamental physics, the study of non-redox proteins offers broad application prospects. Their exceptional sensitivity to minute environmental perturbations (e.g., antigen/ligand binding, pH fluctuations) confers an inherent advantage for constructing high-signal-to-noise, label-free biosensors [8]. Furthermore, resolving the characteristic conductance fingerprints of amino acids and structural motifs serves as the foundational principle for emerging technologies like single-molecule protein sequencing.

**Figure 3:**
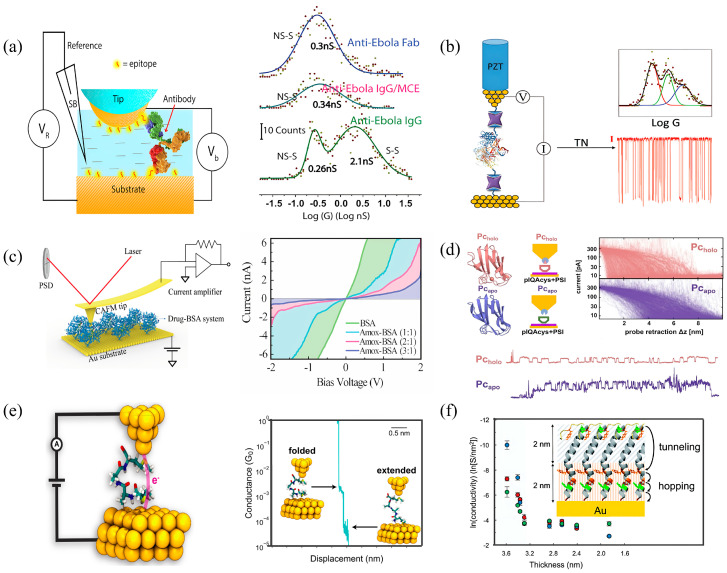
Conductance in non-redox proteins. (**a**) Schematic of partial antibody conductance measurement via EC-STM (left) and its conductance distribution histogram (right). (**b**) Schematic of polymerase conductance measurement via EC-STM (left), along with its conductance distribution histogram and current-time (I-t) trace (right). (**c**) Schematic of BSA conductance measurement via CP-AFM (left) and its current-voltage (I-V) trace (right). (**d**) Schematics of holo- and apo-proteins, accompanied by 2D conductance distributions and (I-t) traces. (**e**) Schematic of peptide conductance measurement via STM-BJ (left) and associated 2D histogram (right). (**f**) Schematic of pilus conductance measurement and the inset illustrates its conductance mechanism. Panel (**a**) is adapted from ref. [8], ACS. Panel (**b**) is adapted from ref. [104], ACS. Panel (**c**) is adapted from ref. [105], MDPI. Panel (**d**) is adapted from ref. [106], ACS. Panel (**e**) is adapted from ref. [11], PNAS. Panel (**f**) is adapted from ref. [107], RSC.

**Figure 4:**
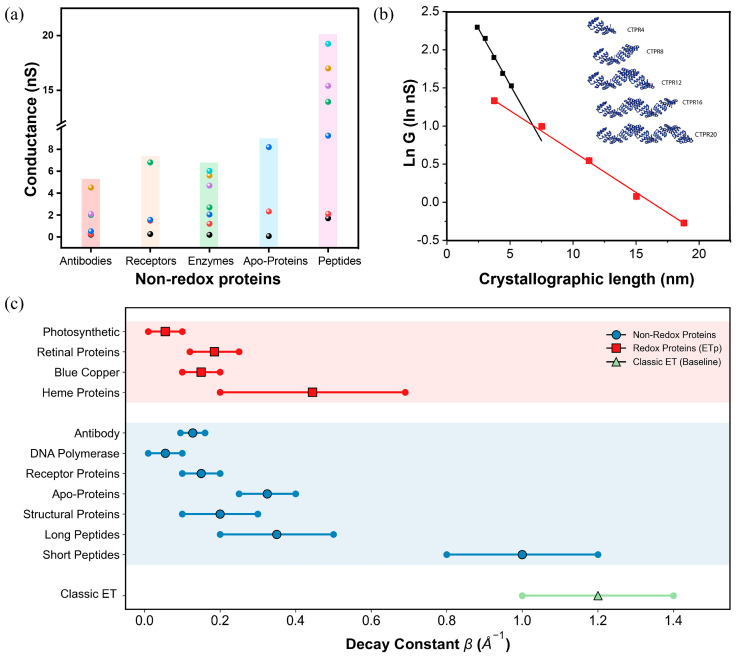
Comparison of conductance values among non-redox proteins. (**a**) Conductance statistics for various non-redox proteins; each point represents a single conductance value. (**b**) Conductance decay fitted to an exponential with a decay constant *1*/*λ* = 0.107 ± 0.003 nm^−1^, the inset illustrates CTPR nanowires synthesized with N- and C-terminal cysteines, depicting structural projections perpendicular to the long axis. Panel (**b**) is adapted from ref. [6], ACS. (**c**) A statistical contrast of the decay constants of non-redox and redox proteins. For data sources of non-redox proteins, see Table 3; for redox proteins, see the ref. [17].

Consequently, research into non-redox protein conductance represents not merely a physical extension of charge transport studies but an essential pathway toward next-generation proteomics and diagnostic devices. This section systematically reviews recent advances in the field, categorizing findings by protein type and structural complexity to build a coherent picture of the structure–conductance–function relationships in non-redox systems (Figure 3 and Figure 4 and Table 3).

### 4.1. Amino Acids and Peptides

Amino acids and short peptides serve as the foundational units and highly tunable model systems for protein electronics. Their charge transport properties emerge from the intricate interplay of side-chain chemistry, backbone conformation, and dynamic response to environmental stimuli, offering a simplified framework to decipher more complex protein behavior (Figure 3e) [11].

The electronic character is fundamentally governed by side-chain chemistry. Studies consistently demonstrate that electron-rich aromatic residues (e.g., Trp, Tyr, Phe) act as efficient mediators [99]. When incorporated into an alanine-based peptide backbone, these residues can enhance single-molecule conductance by 2- to 5-fold via side-chain-mediated tunneling or hopping, following a chemically specific order (Trp > Tyr > Phe > Ala) [21]. The protonation state of residues like tyrosine serves as a powerful chemical gate, where deprotonation alters orbital alignment and strengthens electrode coupling, leading to a further conductance increase [100]. This chemical sensitivity enables functional applications; for instance, the conductance of a polypeptide can be reversibly modulated by changing pH or via specific metal-ion binding, effectively creating a pH- or ion-gated molecular switch.

Beyond side-chain effects, backbone length and conformational dynamics are critical. For very short, rigid peptides (2–4 residues), exceptionally high conductance values (10^−3^ to 10^−4^ *G*_0_) have been measured, classifying them as superior molecular wires [57]. Conductance typically decays exponentially with length (*G ∝ e^−βL^*), but peptides with aromatic stacks exhibit lower decay constants (*β* < 0.6 Å^−1^), supporting more efficient long-range transport [11]. Furthermore, the inherent flexibility of longer peptides leads to conductance polymorphism. During mechanical stretching in a break junction, multiple discrete conductance plateaus are observed, corresponding to different transient folding states where internal hydrogen-bond networks reconfigure to modulate the connectivity of electron transport pathways [11].

In the study of single-molecule conductance, a comparative analysis of the distance decay constants (*β*) between non-redox and redox proteins reveals a mechanistic convergence (Figure 4c) [8]. In classical biological electron transfer (ET), redox proteins are constrained by coherent tunneling between specific cofactors and exhibit a steep distance dependence (*β* ≈ 1.0–1.4 Å^−1^). However, under the applied bias of a single-molecule junction, these proteins utilize their redox centers as efficient hopping intermediates, which drastically reduces the decay constant (*β* ≈ 0.1–0.3 Å^−1^). Surprisingly, recent measurements demonstrate that large non-redox proteins, despite a complete absence of metallic or organic electron transfer centers, exhibit remarkably similar low *β* values (≈0.1–0.2 Å^−1^). This unexpected similarity underscores a profound physical insight: the protein backbone and its intrinsic hydrogen-bond network are inherently capable of supporting efficient, long-range multistep hopping charge transport. This finding challenges the conventional assumption that specific redox cofactors are essential for achieving high conductance in nanoscale proteins [13].

Within these chiral peptide structures, electron transport can exhibit quantum mechanical phenomena such as Chiral-Induced Spin Selectivity (CISS) [101,102]. Experiments on α-helical peptides have demonstrated spin polarization rates exceeding 60%, where the direction of the peptide’s intrinsic electric dipole controls the orientation of spin-polarized current. This establishes chiral peptides as potential tunable spin filters for molecular spintronics.

Finally, the distinct electronic signatures of amino acids can be harnessed for analytical applications. Recognition tunneling, which employs functionalized electrodes to capture single molecules, allows for the distinction of different amino acid side chains and even D/L enantiomers by analyzing the characteristic telegraph noise in the tunneling current [103]. When combined with machine learning for pattern recognition, this technique provides a viable physical basis for the ultimate goal of enzyme-free, single-molecule protein sequencing.

### 4.2. Signal and Recognition Proteins

#### 4.2.1. Antibodies

As naturally bivalent, Y-shaped non-redox proteins, immunoglobulins (e.g., IgG and IgE) serve as ideal model systems for elucidating the impact of contact geometry on electron transport. A series of our studies on IgE and IgG revealed a profound dependence of conductance on specific biological binding (Figure 3a) [15]. Antibody conductance exhibits a bimodal distribution governed by contact topology. A low-conductance state (~0.3 nS) corresponds to non-specific adsorption, where conductance decays with electrode separation due to contact slippage. Conversely, a high-conductance state (~2.2 nS) emerges exclusively when both Fab arms specifically bind to antigens (the S-S mode). This bispecific anchoring establishes a rigid molecular bridge that exhibits rare distance-independent conductance upon stretching, confirming that transport is limited by the contact interface rather than the protein bulk. Mechanistically, this high-conductance state is attributed to a hybrid model combining tunneling injection and hopping, characterized by an exceptionally low decay constant (*β* ≈ 0.16 nm^−1^) and a distinct resonance peak near +300 mV (vs. NHE).

#### 4.2.2. Receptor Proteins

Transmembrane receptors like Integrin α_v_β_3_ (a 200 kD non-redox protein) demonstrate the decisive role of receptor–ligand interactions in gating long-range electron channels. Zhang et al. showed that specific binding of RGD ligands acts as a gatekeeper for overcoming charge injection barriers [71]. Under single-sided specific binding, α_v_β_3_ exhibited a conductance peak of ~0.38 nS and sustained giant currents (>0.1 nA) over distances > 3 nm. In contrast, homologous controls (α_4_β_1_) lacking the binding site showed negligible signals [60]. Physically, integrins display unique non-equilibrium fluctuations. Under bias, they manifest characteristic telegraph noise with “ON” states persisting for milliseconds, attributed to dynamic instabilities in hydrogen bond networks. Electronic structure calculations suggest that the energy level spacing follows “quantum criticality” statistics—intermediate between metals and insulators—implying that thermal fluctuations may drive phase transitions between localized and band-like transport states.

Additionally, Bacteriorhodopsin (bR) offers a complementary paradigm for cofactor-mediated transport in transmembrane systems. Although physiologically a proton pump, bR exhibits current densities four orders of magnitude higher than lipid bilayers in solid-state junctions, with temperature-independent transport (80–300 K) [3]. This supports a Quantum Tunneling model, suggesting that the internal conjugated Retinal cofactor acts as a critical potential well, significantly lowering the energy barrier for electron traversal even in the absence of metal centers.

#### 4.2.3. Streptavidin

Streptavidin (SA) is a benchmark model due to its high stability and specific biotin binding [108]. However, interpretations of its conductance vary significantly by technique, highlighting the distinction between single-molecule and ensemble physics. STM studies demonstrate that specific biotin–streptavidin interactions enhance conductance to ~6.8 nS compared to non-specific surface linkages (~0.36 nS). Energetically, a characteristic conductance resonance near +300 mV (vs. NHE) is observed, which is about 0.7 V lower than the solution oxidation potential of its aromatic residues [8]. This indicates the protein matrix drastically reduces reorganization energy, enabling efficient resonant tunneling through discrete molecular orbitals.

In contrast, ensemble measurements using EGaIn and CP-AFM found that biotin binding has a negligible impact on the bulk transport efficiency of SA monolayers [79]. This discrepancy suggests that the switching observed in STM likely originates from contact mechanics, wherein biotin binding creates a mechanically stiffer junction that improves coupling, rather than from a fundamental change in the protein’s bulk electronic properties. Furthermore, Bera et al. noted that while biotin induces structural tightening (e.g., β-barrel contraction), the electron transport pathway likely does not traverse the region most perturbed by binding [79]. This serves as a cautionary note: when designing conformational biosensors, the spatial overlap between the electron pathway and the binding site is critical.

### 4.3. Catalytic Proteins

#### 4.3.1. DNA Polymerase

Studies on non-redox Φ29 DNA polymerase demonstrate how interface engineering enables electronic interrogation of enzymatic function [8]. Zhang et al. addressed this by genetically engineering dual biotin tags at domains distal to the active site (N-terminus and exonuclease), enabling robust dual-point anchoring via streptavidin–biotin links (Figure 3b). Compared to single-point or nonspecific attachment (~0.2 nS), dual anchoring yields high conductance (~5.6 nS), accessing the hydrophobic core. This state confers exceptional conformational sensitivity: open-to-closed transitions shift DC conductance, while catalytic motions generate high-amplitude telegraph noise (>25% fluctuation), enabling real-time readout of enzymatic kinetics.

Mechanistically, Φ29 mirrors streptavidin with a shared resonance peak at ~300 mV (vs. NHE), indicating aromatic amino acid networks mediate universal transport in non-redox proteins. Potential tuning to align injection with resonance thus optimizes sensitivity for monitoring enzymatic activity [53].

#### 4.3.2. Proteasome

Extending single-molecule conductance measurements to large multi-subunit enzymes is a key frontier in bioelectronics. Afsari et al. recently characterized the liquid-phase conductance of the 20S proteasome core particle (~700 kD) using STM [52]. The wild-type CP exhibits robust conductance (~2–3 nS) mediated by a dense network of aromatic residues (Trp, Tyr, Phe) with edge-to-edge distances < 1.2 nm, satisfying criteria for efficient hopping. Crucially, conductance is sensitive to the loading state. While small peptide substrates induce minimal change, the entry of unfolded macromolecular substrates into the central pore of the Δ12 mutant causes a precipitous drop in conductance. This “signal blocking” effect likely results from steric occlusion of the aromatic network or dielectric disruption, establishing a new paradigm for monitoring macromolecular processing.

### 4.4. Structural and Apo-Proteins

#### Structural Insights from Serum Albumin, CTPR and Apo-Proteins

Studies on bovine serum albumin (BSA) establish a direct link between secondary structure and conductance. Yu et al. found that drug-induced unraveling of α-helix into random coils or β-sheets correlates with a monotonic decay in conductance (Figure 3c) [105]. This supports the “Hopping Transport” theory: the compact axial spacing of residues in α-helices (0.15 nm) facilitates orbital overlap better than the expanded spacing in β-sheets (0.32–0.34 nm). This identifies the α-helix as a preferred conductive pathway, providing a basis for detecting conformational diseases (e.g., Alzheimer’s).

Engineered CTPR proteins, with their rigid helix-turn-helix repeats, provide a tunable platform to isolate the effects of length and structure [6]. They exhibit extraordinary long-range conductivity with very slow conductance decay beyond 6 nm, outperforming many synthetic molecular wires (Figure 4b). Their transport mechanism is identified as incoherent hopping, evidenced by a resistance that scales with the square of the length (*R* ∝ *L*^2^) under low bias. This efficiency originates from a unique dielectric environment: the rigid hydrophobic core shields charge carriers from solvent, drastically reducing reorganization energy. This is confirmed by a conductance resonance at a similarly lowered potential (~300 mV vs. NHE), demonstrating how protein architecture can create electron-transparent pathways [8].

A pivotal strategy for deconvoluting backbone conductivity from prosthetic group effects is the comparison of Holo- and Apo-proteins. The study of apo-proteins (with cofactors removed) is decisive for isolating the polypeptide backbone’s contribution (Figure 3d). Amdursky et al. demonstrated that removing the metal center (Apo-form) triggers a mechanistic transition [109]. While Holo-proteins exhibit temperature-independent coherent tunneling, Apo-proteins transition to thermally activated incoherent hopping (T > 200 K). This confirms that the polypeptide backbone is inherently capable of charge transport, but the metal ion acts as a gain element, facilitating efficient tunneling by providing resonance levels or minimizing reorganization energy [9]. In contrast, bR relies on its conjugated retinal cofactor. Removal of retinal (Opsin) causes a precipitous drop in conductivity, which is fully restored upon reconstitution. This identifies the conjugated cofactor, rather than the protein backbone, as the primary molecular wire in this system.

### 4.5. Microbial Nanowires (e-pili)

Microbial electrically conductive pili (e-pili), particularly Type IV pili produced by *Geobacter* species, represent a remarkable evolutionary adaptation for extracellular electron transport (Figure 3f) [110]. The consensus mechanism attributes their conductivity to π-π stacking of aromatic residues (primarily phenylalanine and tyrosine) along the helical axis, enabling hopping or delocalized charge transport [111]. This is exemplified by the “metal-like” conductivity observed in *G. metallireducens* pili, which feature exceptionally high aromatic density, in contrast to the lower conductivity of *G. sulfurreducens* pili.

Resolving longstanding debates on nanowire identity, Liu et al. used AFM-IR and immunogold labeling to demonstrate that *G. sulfurreducens* secretes distinct, cytochrome-free PilA filaments (~3 nm diameter) with intrinsic conductivity [112]. The current scientific consensus recognizes PilA-based aromatic hopping pathways and multi-heme cytochrome wires (e.g., OmcS) as coexisting, complementary systems for electron transfer. As programmable bioelectronic materials, e-pili offer tunable conductivity through genetic engineering (e.g., tryptophan insertion), supramolecular assembly into nanobundles, or environmental modulation (e.g., pH-induced conformational stiffening), providing a versatile blueprint for sustainable bio-energy devices and sensors [113].

**Table 3:** Summary of Conductance Values for Different Non-Redox Proteins.

Category	Name	Detection Technology	Conductance	*β* Value	Ref.
Amino Acids and Peptides	Cys-Gly-Cy	STM-BJ	~19 nS	~0.87 Å^−1^	[114]
	Phe-Trp-Cys-Gly	STM-BJ	~10 nS	N.A.	[115]
	4Ala	MCBJ	~78 nS	~1.35 Å^−1^	[57]
	Aromatic foldamers	STM-BJ	~7.7 × 10^3^ nS	~0.02 Å^−1^	[116]
Antibody	IgG & IgG-Fab	EC-STM	~0.2–2 nS	~0.016 Å^−1^	[71]
	IgE	STM	~0.2 nS	~0.01 Å^−1^	[15]
Receptor proteins	Integrin α_v_β_3_	STM	~0.38 nS	N.A.	[97]
	Bacteriorhodopsin	STM	~1.7 nS	N.A.	[117]
	Streptavidin	QMT probes	~1.5–25 nS	N.A.	[60,72]
	Streptavidin	STM	~0.2–6.8 nS	N.A.	[8,71,79]
DNA Polymerase	Φ29 polymerase	STM	~0.2–5.6 nS	N.A.	[104]
	Human DNA polymerases β	G-M-G junction	~600 nS	N.A.	[118]
Proteasome	20S proteasome	STM	~2–3 nS	N.A.	[52]
	Δ12 Proteasome Mutant	STM	~6 nS	N.A.	
Structural Proteins	Bovine Serum Albumi	CP-AFM	~3 nS	N.A.	[105]
	CTPR (4–20)	STM	~0.7–3.7 nS	N.A.	[6]
	CTPR8	CP-AFM & STM	~2 nS	~0.01 Å^−1^	[13,69]
Apo-Proteins	Apo-azurin	CP-AFM	~0.2–2 nS	N.A.	[68]
	Zn-Azurin	ECSTM	N.A.	~0.4 Å^−1^	[119]
Microbial Nanowires (e-pili)	*Geobacter* *sulfurreducens* pili	Nano-electrode platform	~51 mS/cm	N.A.	[120]
	*G.metallireducens* pili	Nano-electrode platform	~277 S/cm	N.A.	[113]
	A80W A109W pili	CP-AFM	~43 mS/cm	N.A.	[121]
	*G.sulfurreducens* e-pili	CP-AFM	~1.4–4.3 S/cm	N.A.	[122]

## 5. Applications for Non-Redox Protein Conductance

**Figure 5:**
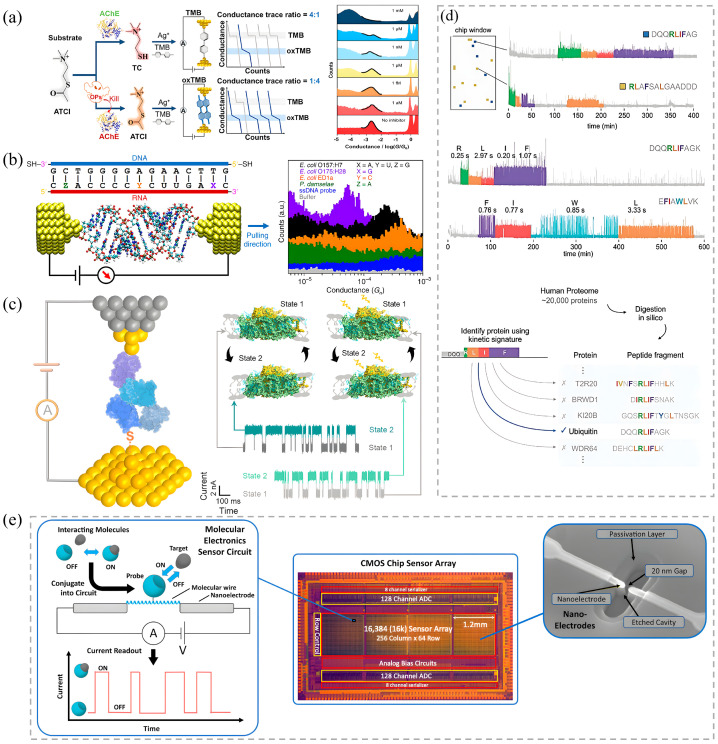
Applications of non-redox protein conductance. (**a**) Enzyme-mediated detection of methamidophos (MTMP) via conductance trace ratio analysis. (**b**) STM-BJ detection of mRNA hybridization to a DNA probe, showing 2D conductance distributions. (**c**) Real-time protein conformation detection via current-time (I-t) traces. (**d**) Discrimination of four nucleobases: schematic (left), I-t curves (middle), and histograms (right). (**e**) Molecular Electronic Sensor Concept: A synthetic α-helical protein wire bridging nanoelectrodes acts as a probe (left). These sensors are integrated into a large-scale CMOS array (middle). SEM image shows the ~ 20 nm nanogap electrodes (right). (Panel (**a**) adapted from Ref. [127], ACS; (**b**) from Ref. [84], Springer Nature; (**c**) from Ref. [130], Springer Nature; (**d**) from Ref. [61], AAAS; (**e**) from Ref. [93], PNAS).

The discovery of intrinsic conductivity in non-redox proteins has fundamentally broadened the scope of bioelectronics, shifting the paradigm from a niche focus on metalloproteins to the potential utilization of the entire proteome. This breakthrough implies that diverse functional biomolecules can be integrated into solid-state architectures as active electronic elements. Research has firmly established that these proteins support measurable, long-range charge transport via specific interfacial contacts and internal hopping or tunneling pathways [8]. Importantly, their conductive states are highly sensitive modulators of biological function—responsive to ligand binding, conformational dynamics, and environmental stimuli. This unique confluence of electronic functionality and biological specificity is propelling the field from fundamental exploration toward the development of transformative devices. Emerging applications in single-molecule sequencing, label-free biosensing, drug interaction kinetics, and bio-spintronics demonstrate a potential that could surpass conventional materials (Figure 5). However, translating this potential into robust, high-performance devices requires overcoming inherent challenges such as Debye screening in physiological buffers and the stochastic noise characteristic of soft-matter interfaces [71].

### 5.1. Next-Generation Single-Molecule Sequencing

Transducing the mechanical motion of enzymes into electrical signals represents the most promising frontier in this field. Unlike traditional sequencing technologies relying on optical labeling or ionic current blockage, conductance-based methods utilize the enzyme itself as a direct electromechanical transducer.

DNA/RNA Sequencing: The DNA polymerase Φ29 exemplifies this approach. Integrated into a nanojunction, the enzyme acts as a sensitive reporter. The conformational motions associated with dNTP capture and active-site closure during DNA synthesis directly modulate the junction current, producing characteristic millisecond-scale fluctuations [123]. By integrating such single-molecule junctions into high-density CMOS arrays containing thousands of parallel sensors, this electronic readout offers a viable alternative to optical methods, avoiding issues like photobleaching [93]. Its non-invasive nature allows for the capture of subtle kinetic details, enabling the distinction of features like single-nucleotide polymorphisms (SNPs) in RNA:DNA hybrids (Figure 5b,e).

Protein Sequencing: For de novo protein sequencing, a significant challenge is the rapid diffusion of small analytes (Figure 5d) [124]. Recognition tunneling (RT) spectroscopy addresses this by functionalizing electrodes with specific reader molecules that temporarily trap target amino acids or peptides through non-covalent interactions [103]. This technique amplifies and distinguishes the tunneling current fingerprints of all 20 natural amino acids, including structural isomers like leucine/isoleucine and post-translational modifications such as methylation, a notable advantage over conventional mass spectrometry at the single-molecule level. A complementary strategy exploits natural degradation machinery; studies on the 20S proteasome show that peptide translocation through its channel produces conductance blockades correlated with substrate sequence. The future success of these sequencing platforms hinges on the integration of advanced machine learning algorithms to deconvolve the complex, stochastic signal patterns and on overcoming the Debye screening limit, possibly by employing engineered short-range sensing architectures or unfolded polypeptides [81].

### 5.2. Ultrasensitive Label-Free Biosensing

Non-redox protein sensors exploit a unique “contact-dependence” mechanism that endows them with exceptional signal-to-noise ratios (SNR) [8]. The high SNR arises not merely because the protein becomes conductive, but because specific ligand–receptor binding (e.g., Antibody–Antigen) establishes a mechanically rigid molecular bridge. In contrast, non-specific adsorption results in loose, fluctuating contacts that fail to support stable tunneling currents. This inherent mechanochemical selectivity allows for the specific electrical capture of target proteins and effectively filters out background noise.

Clinical Diagnostics: Researchers have achieved the specific electrical capture of biomarkers such as Ebola virus, HIV antibodies, and integrins [125,126]. To overcome the diffusion limit at ultralow concentrations, Dielectrophoresis (DEP) technology has been introduced to concentrate trace molecules into the nanogap via AC electric fields. This combined capture-and-measure strategy has pushed detection limits to the attomolar (10^−18^ M) range, far surpassing traditional assays like ELISA.

Environmental Monitoring: This versatility extends to environmental safety. Dong et al. constructed a sensor based on Acetylcholinesterase (AChE) inhibition for detecting organophosphorus pesticides (Figure 5a) [127]. The irreversible binding of pesticide molecules blocks the conductive network, leading to a precipitous current drop. This method achieved a 10 aM detection limit in natural water, demonstrating the robust anti-interference capability of non-redox proteins in complex matrices.

### 5.3. Pharmacokinetic Screening and Interaction Kinetics

In pharmaceutical discovery, single-molecule conductance provides a powerful tool for label-free, real-time kinetics analysis. Drug binding often induces conformational stabilization or allosteric changes in the target protein, which in turn modulates its electronic pathway (Figure 5c) [128]. Unlike ensemble methods that typically yield only an equilibrium dissociation constant (*K_D_*), this technology records the full trajectory of binding and dissociation events. By analyzing the frequency and duration of characteristic current fluctuations, researchers can directly extract the association (*k_on_*) and dissociation (*k_off_*) rate constants.

This capability is particularly valuable for identifying drugs with long residence times, a key predictor of in vivo efficacy. For example, studies on the interaction between BSA and the drug warfarin showed that drug binding increases α-helical rigidity, leading to a detectable conductance decrease [105]. This approach offers a new, information-rich physical parameter for high-throughput screening of molecular interactions.

### 5.4. Biomolecular Electronics and Spintronic Devices

Beyond sensing, non-redox proteins are actively being developed as functional materials for biocompatible nanocircuits and quantum devices.

Bio-Molecular Wires: To address the scalability challenges of molecular electronics, proteins like CTPR have been designed as tunable molecular wires with precise lengths from 4 to 20 nm [6]. These wires exhibit exceptionally slow conductance decay over distance due to efficient hopping through aromatic networks and low reorganization energy, outperforming many synthetic organic molecules and mitigating interconnection losses in nanoscale circuits.

Bio-Spintronics: The inherent chirality of protein structures, particularly α-helices, gives rise to the CISS effect, where electron transport becomes spin-polarized [129]. Recent breakthroughs demonstrate a dipole-induced spin reversal, where flipping the peptide’s intrinsic electric dipole moment controls the direction of spin polarization [101]. This reveals that non-redox proteins can function as room-temperature spin valves or quantum memory elements without external magnetic fields, offering a biomolecular pathway for the hardware of future spintronic and quantum information technologies.

In summary, applied research on non-redox protein conductance is successfully bridging fundamental science and prototype development. By harnessing the specificity of biological recognition, the dynamics of conformational change, and the quantum effects of chiral structures, this field is providing revolutionary tools for life science analysis and illuminating a path of bio-abiotic fusion for next-generation miniaturization and multi-functionalization electronics.

## 6. Challenges and Future Outlook

**Figure 6:**
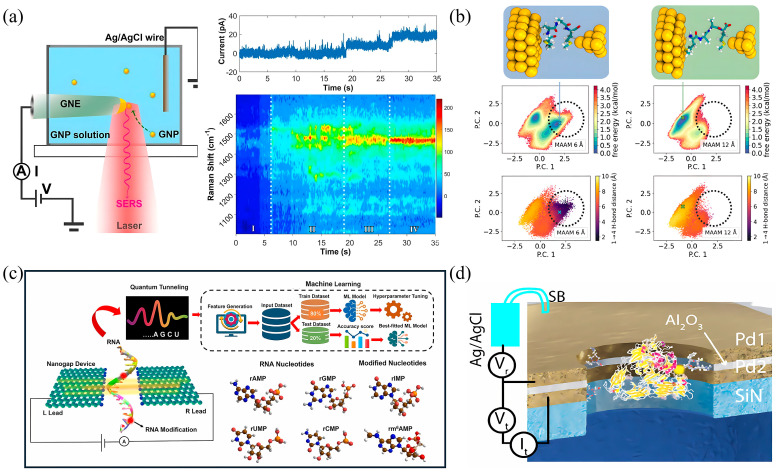
Emerging technologies for analyzing protein conductance. (**a**) Real-time monitoring of single-nanoparticle collisions, correlating I-t traces with Raman spectra. (**b**) Workflow of machine learning-assisted quantum tunneling for high-throughput RNA sequencing. (**c**) Computation of dominant molecular conformations using Non-Equilibrium Green’s Function-Density Functional Theory (NEGF-DFT). (**d**) Schematic of an AI-driven Multi-Step Pulse Coding (MPC) algorithm for signal segmentation. (Panel (**a**) adapted from Ref. [133], ACS; (**b**) from Ref. [11], PNAS; (**c**) from Ref. [134], ACS; (**d**) from Ref. [135], IOP.)

Significant progress in single-molecule conductance of non-redox proteins has established their potential as bioelectronic components. However, the field faces substantial hurdles in transitioning from phenomenon discovery to mechanistic validation and engineering application. To facilitate the leap from fundamental physics to reliable bioelectronic devices, we delineate four core challenges currently impeding progress and propose corresponding frontier pathways for future development (Figure 6).

### 6.1. Challenges

#### 6.1.1. Conformational Heterogeneity and Signal Convolution

Under physiological conditions, proteins do not exist as static crystal structures but rather as dynamic conformational ensembles [128]. This intrinsic dynamism renders the interpretation of conductance signals exceptionally complex, primarily resulting in the convolution of intrinsic biological signals with experimental noise.

Single-molecule experiments (e.g., STM-BJ) typically generate broad conductance histograms. A persistent challenge lies in distinguishing whether this dispersion stems from measurement artifacts (e.g., variations in contact geometry) or genuinely reflects the protein’s intrinsic structural landscape [11]. While secondary structures (α-helices vs. β-sheets) and side-chain packing significantly modulate transport, deconvolving the contribution of specific conformations from broad statistical distributions remains a formidable task. Moreover, it is difficult to distinguish function-related conformational switching. The lack of detection methods capable of simultaneously capturing transient conformations and transient currents limits the precise elucidation of structure–conductance relationships.

#### 6.1.2. The Bio-Abiotic Interface Instability

Constructing stable “electrode-protein-electrode” junctions is the prerequisite for reliable measurement. However, the heterogeneous interface between soft biomolecules and hard inorganic electrodes represents the weakest link in the system. Direct physical adsorption of proteins onto bare metal surfaces often leads to irreversible conformational rearrangement or denaturation, causing the loss of both biological activity and intrinsic conductivity [131].

In probe-based techniques, contact formation is typically stochastic, lacking atomic-level control over molecular orientation. Furthermore, in the absence of efficient coupling linkers, substantial Schottky barriers often exist at the interface, meaning measurement results frequently reflect contact resistance rather than the bulk conductance of the protein [45]. Furthermore, weak non-covalent interactions struggle to withstand high electric fields and mechanical stress, resulting in extremely short junction lifetimes [60]. This instability precludes long-term dynamic monitoring and the analysis of low-frequency kinetic signals.

#### 6.1.3. The Gap Between Experiment and Theory

Validating proposed mechanisms, such as tunneling, hopping, or flickering resonance, faces a dual bottleneck. From an experimental perspective, the gold standard for distinguishing transport mechanisms is temperature-dependent measurements. However, conducting broad-temperature-range scans in liquid environments involves significant technical challenges, including solvent evaporation, thermal drift, and the narrow thermal stability window of biological samples. These factors make experimental validation using SPM techniques particularly demanding [22]. Theoretically, modeling non-redox proteins requires explicit solvent representations, which impose prohibitive computational costs for high-level ab initio calculations. Moreover, existing simplified models often neglect quantum coherence or dynamic side-chain effects, resulting in substantial discrepancies between theoretical predictions and experimental observations [132].

#### 6.1.4. Scalability and Device Reproducibility

Current research is largely confined to specialized, low-throughput laboratory setups, making translation to practical devices difficult. Due to conformational heterogeneity and random interfacial contacts, device-to-device variability is extreme. Furthermore, effective methods to scale the superior electrical properties observed at the single-molecule level to macroscopic protein films or nanowire arrays are still lacking. Achieving synergistic signal amplification while maintaining microscopic structural order remains a key bottleneck.

### 6.2. Future Outlook

#### 6.2.1. Development of Multimodal Measurement Techniques

To establish causality between conformation and conductance, future platforms will likely require structure–electrical synchronization capabilities. One promising direction is optoelectronic coupling, where conductance measurements are integrated with techniques such as Surface-Enhanced Raman Spectroscopy (SERS) or single-molecule Fluorescence Resonance Energy Transfer (smFRET) (Figure 6a). SERS enables monitoring of ligand-induced chemical fingerprint changes, and FRET tracks domain conformational dynamics, allowing both signals to be precisely time-correlated with conductance measurements [136]. Similarly, correlative microscopy combining High-Speed Atomic Force Microscopy (HS-AFM) with conductive probes allows for direct correlation of dynamic morphological changes, such as enzymatic gating, with electrical responses on millisecond timescales, embodying a “see it as you measure it” paradigm [137]. On the theoretical front, advanced multiscale models integrating quantum-chemical calculations, molecular dynamics (MD), non-equilibrium Green’s function (NEGF) methods (Figure 6b), and machine learning approaches are essential. Such models can capture atomic structure, conformational dynamics, solvent effects, and quantum coherence, providing a comprehensive framework to bridge the gap between experiment and theory.

#### 6.2.2. Precision Interface Engineering

To address contact instability, future interface strategies are expected to shift from random physical adsorption toward precise chemical design. One promising approach involves site-specific anchoring, which employs techniques such as unnatural amino acid incorporation or click chemistry to introduce robust anchoring groups (e.g., thiols or diazonium salts) at defined rigid domains, thereby ensuring consistent molecular orientation and stable coupling [138]. In parallel, the development of soft contacts using biocompatible conductive hydrogels or polymer electrodes is critical for preserving native protein structure by mimicking the physiological dielectric environment and minimizing impedance mismatch at the bio–abiotic interface.

#### 6.2.3. AI-Driven Multiscale Theoretical Modeling

Artificial intelligence is poised to play an increasingly critical role in overcoming the computational scaling limits inherent in simulating bioelectronic systems (Figure 6c). One key advancement lies in Machine Learning Force Fields (MLFF), where algorithms trained on quantum-chemical data can accelerate Molecular Dynamics (MD) simulations by orders of magnitude [139]. This enables the capture of rare, long-timescale conformational events, such as those driving flickering resonance. Complementing this, deep learning models trained on combined experimental and theoretical datasets may soon enable sequence-to-conductance prediction, allowing for the rapid screening of conductive motifs directly from amino acid sequences without relying solely on computationally expensive quantum chemical calculations.

#### 6.2.4. Synthetic Biology and Integrated Devices

To address the challenge of scalability, the field is likely to evolve from merely “reading” natural proteins toward the deliberate “designing” of electronic proteins. One promising avenue is de novo design [140], where synthetic biology provides the toolset to engineer proteins with ultra-high thermal stability and optimized electron pathways, such as aligned aromatic channels. To realize the potential of such designs, parallel advances in CMOS integration are essential. The development of semiconductor-based nanoelectrode arrays will enable massively parallel measurements (Figure 6d), allowing statistical averaging to overcome individual stochasticity [93]. Ultimately, the creation of organic-inorganic hybrid systems, such as protein–carbon nanotube hybrids, can pave the way for bioelectronic devices that combine the inherent sensitivity of biological molecules with the reliability required for industrial-scale applications.

## 7. Summary

This review synthesizes the rapidly evolving landscape of non-redox protein conductance, charting its trajectory from fundamental mechanistic inquiries to cutting-edge bioelectronic applications. Taken together, this work shows that non-redox proteins are far from the electrical insulators they were long assumed to be. Instead, a compelling body of evidence from diverse systems, including antibodies, receptors, enzymes, and peptides, demonstrates that these biomolecules function as sophisticated, tunable electronic components capable of efficient charge transport.

Mechanistically, the field has transcended simple tunneling models to embrace a nuanced framework where charge transport is governed by the interplay of coherent tunneling, incoherent hopping mediated by aromatic networks, and dynamic flickering resonance effects. A central theme emerging from these studies is the concept of conformation-conductance coupling, where electron transport efficiency is intimately modulated by the protein’s dynamic structural state, secondary structure integrity, and interfacial contact geometry.

Technologically, measurement techniques like STM-based break junctions, CP-AFM, and integrated semiconductor platforms have advanced to enable precise probing of these properties, revealing conformational sensitivity and contact-dependent gating. These tools have facilitated applications in label-free biosensing, enzymatic kinetics monitoring, drug screening, and single-molecule sequencing.

Despite these achievements, challenges persist in decoupling conformational noise from intrinsic signals, stabilizing bio-abiotic interfaces, validating mechanisms across scales, and achieving device reproducibility. Future progress will depend on multimodal correlative methods, precision interface engineering, AI-enhanced modeling, and synthetic protein design integrated with scalable platforms.

In summary, conductance in non-redox proteins suggests a link between biophysics and electronics. This connection offers a new way to think about biological charge transfer and points toward the development of biocompatible devices with interesting possibilities for specificity and function.

## Data Availability

Not applicable.
